# Comparative study of the effects of different radiation qualities on normal human breast cells

**DOI:** 10.1186/s13014-017-0895-8

**Published:** 2017-09-25

**Authors:** Dajana Juerß, Monique Zwar, Ulrich Giesen, Ralf Nolte, Stephan Kriesen, Giorgio Baiocco, Monika Puchalska, Marc-Jan van Goethem, Katrin Manda, Guido Hildebrandt

**Affiliations:** 1 Department of Radiotherapy and Radiation Oncology, University Medical Centre Rostock, Suedring 75, 18059 Rostock, Germany; 2 Physikalisch-Technische Bundesanstalt (PTB), Bundesallee 100, 38116 Braunschweig, Germany; 3 Physics Department, University of Pavia, Via Bassi 6, 27100 Pavia, Italy; 4 Technische Universität Wien, Atominstitut, Stadionallee 2, 1020 Vienna, Austria; 5 KVI - Center for Advanced Radiation Technology (KVI-CART), Zernikelaan 25, 9747 AA Groningen, The Netherlands

**Keywords:** Neutron irradiation, X-rays, Normal human breast cells, Relative biological effectiveness

## Abstract

**Background:**

As there is a growing number of long-term cancer survivors, the incidence of carcinogenesis as a late effect of radiotherapy is getting more and more into the focus. The risk for the development of secondary malignant neoplasms might be significantly increased due to exposure of healthy tissue outside of the target field to secondary neutrons, in particular in proton therapy. Thus far, the radiobiological effects of these neutrons and a comparison with photons on normal breast cells have not been sufficiently characterised.

**Methods:**

MCF10A cells were irradiated with doses of up to 2 Gy with neutrons of different energy spectra and X-rays for comparison. The biological effects of neutrons with a broad energy distribution (<*E*_n_ > = 5.8 MeV), monoenergetic neutrons (1.2 MeV, 0.56 MeV) and of the mixed field of gamma’s and secondary neutrons (<*E*_n_ > = 70.5 MeV) produced by 190 MeV protons impinging on a water phantom, were analysed. The clonogenic survival and the DNA repair capacity were determined and values of relative biological effectiveness were compared. Furthermore, the influence of radiation on the sphere formation was observed to examine the radiation response of the potential fraction of stem like cells within the MCF10A cell population.

**Results:**

X-rays and neutrons caused dose-dependent decreases of survival fractions after irradiations with up to 2 Gy. Monoenergetic neutrons with an energy of 0.56 MeV had a higher effectiveness on the survival fraction with respect to neutrons with higher energies and to the mixed gamma - secondary neutron field induced by proton interactions in water. Similar effects were observed for the DNA repair capacity after exposure to ionising radiation (IR). Both experimental endpoints provided comparable values of the relative biological effectiveness. Significant changes in the sphere formation were notable following the various radiation qualities.

**Conclusion:**

The present study compared the radiation response of MCF10A cells after IR with neutrons and photons. For the first time it was shown that monoenergetic neutrons with energies around 1 MeV have stronger radiobiological effects on normal human breast cells with respect to X rays, to neutrons with a broad energy distribution (<*E*_n_ > = 5.8 MeV), and to the mixed gamma - secondary neutron field given by interactions of 190 MeV protons in water. The results of the present study are highly relevant for further investigations of radiation-induced carcinogenesis and are very important in perspective for a better risk assessment after secondary neutron exposure in the field of conventional and proton radiotherapy.

**Electronic supplementary material:**

The online version of this article (10.1186/s13014-017-0895-8) contains supplementary material, which is available to authorized users.

## Background

The incidence of carcinogenesis as a late effect of radiotherapy is discussed in several studies as there is a growing number of long-term cancer survivors [1–5]. The risk for the development of secondary malignant neoplasms is significantly increased, especially for breast cancers among women who were irradiated as a treatment for Hodgkin disease in childhood or adolescence using particle proton therapy [6–9], which allows a better dose conformation to the target volume than conventional radiotherapy. Due to a production of secondary neutrons during proton therapy (and also during photon therapy with high energies), healthy tissue distal to the target region can be exposed to neutrons with a potentially high biological effectiveness [10]. Until now, the potential for the induction of neoplasms following low-dose neutron exposures has not been very well characterised.

The DNA can be considered as the most important target of ionising radiation (IR), since misrepair of radiation-induced DNA damage can be the initial step of carcinogenesis. Hence, radiation-induced DNA double-strand breaks (DSBs) were scored 24 h after exposure by visualising and counting residual γH2AX foci, which are considered as a reliable biomarker for the investigation of complex DNA damage [11–13]. The clonogenic survival was determined after neutron and X-ray IR via colony forming assays as a measure of long-term effects and to classify the unknown biological effects of a clinical relevant secondary neutron field into a defined spectrum of different neutron energies. Using X-rays as a reference, the observed radiation effects of neutrons were compared using the concept of relative biological effectiveness (RBE). Irradiations with low-energy monoenergetic neutrons and medium-energy neutrons with a broad energy distribution and a mean energy of about 5.8 MeV were performed. In order to generate a field of secondary neutrons, similar to that produced during proton therapy, a 190 MeV proton beam was directed onto a water phantom.

In 2009, Stingl suggested that “normal stem and progenitor cells are the likely targets for malignant transformation” and have the ability to self-renew [14]. In order to confirm the capacity of healthy mammary cells to function as progenitor cells, and thus as an initial target for carcinogenesis, the present study investigated the self-renewal potential of the MCF10A cells – a non-transformed cell line with properties of progenitor cells [15] – utilising a 3D spheres formation assay as described in literature [14, 16]. Our study of these highly relevant endpoints and measurements of RBE values for neutron exposures will expand on the current knowledge: provided results are useful in perspective for the assessment of the risk of cancer induction by IR, both for radiation protection and for optimization in proton and photon therapy, through the inclusion in treatment planning of the risk evaluation for secondary cancer.

## Methods

### Cell culture

MCF10A (provided by Prof. Kevin Prise, Queen’s University Belfast, Ireland*)*, is a spontaneously transformed cell line from normal human mammary epithelial cells [17], authenticated using STR typing (Additional file 1). The cells were cultivated using Dulbecco’s modified Eagle medium/F12 (DMEM/F12, Gibco/Life Technologies, Darmstadt, Germany) supplemented with 0.01% cholera toxin, 0.1% insulin, 0.05% hydrocortisone and 1% penicillin/streptomycin (all Sigma Aldrich, Hamburg, Germany), 0.02% epidermal growth factor (EGF; Gibco/Life Technologies) and 5% horse serum (Fisher Scientific, Schwerte, Germany) under 5% CO_2_ and at 37 °C. The cells were passaged two times a week.

### Irradiation setup

A homogenous irradiation for cells in a single cell suspension was ensured by using rotating systems. The first setup consists in a ring holder with seven cylindrical containers (1 ml volume, 3 mm thick, 20 mm in diameter), made of polymethylmethacrylate (PMMA), positioned on a wooden motor-driven rotator, which allowed a slow rotation to keep the cells in suspension during the whole irradiation time. This arrangement was used for the irradiations with X-rays, medium-energy neutrons with a broad energy distribution at Physikalisch-Technische Bundesanstalt (PTB, Braunschweig, Germany) and for the mixed gamma - secondary neutron field at the KVI-Center for Advanced Radiation Technology (KVI-CART, Groningen, The Netherlands), where the radiation field was wide enough to perform homogeneous irradiation of more containers at a time with a single dose. For irradiations with low-energy monoenergetic neutrons at PTB the same containers but a modified rotator was used, on which three samples were placed in a row at distances of 50, 70 and 100 mm from the neutron source. This solution allowed to perform irradiation of three containers at a time, each distance corresponding to a different dose point.

Sham irradiated samples were used as negative control. Afterwards the cells were seeded as an adherent culture.

As the reference, photon irradiations with X-rays were performed at ambient temperature using an X-Strahl 200 system (Xstrahl Ltd., Surrey, United Kingdom) at 220 kV, filtered with 1 mm Al, 0.25 mm Cu and 0.45 mm Sn. The dosimetry is based on the German Standard DIN 6809–5. The uncertainties are about ±3%. In addition to irradiations at a high dose rate (HDR) of 0.37 Gy/min, a low dose rate (LDR) of 0.02 Gy/min was used to match the dose rate of the neutron irradiations. Doses of 0.1, 0.25, 0.5, 1, 2, 4 and 6 Gy were used.

**Fig. 1:**
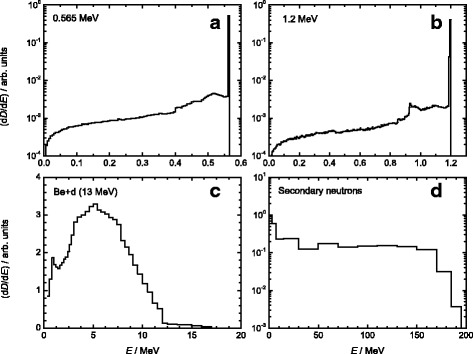
Relative energy distributions of the four different neutron fields. **a** Low-energy monoenergetic neutrons of 0.56 MeV; **b** Low-energy monoenergetic neutrons of 1.2 MeV; **c** Medium-energy neutrons with a broad energy distribution and a mean neutron energy of <*E*_n_ > = 5.8 MeV; **d** Mixed gamma - secondary neutron field with a mean neutron energy of <*E*_n_ > = 70.5 MeV, produced by a 190 MeV proton beam impinging on a water phantom

Two types of neutron irradiations were performed at PTB: firstly, a “medium-energy” intense neutron field with dose rates of 0.1 Gy/min (HDR) and of about 0.003 Gy/min (LDR). It was produced by the ^9^Be + d reaction on a thick Be-target within a collimator at a deuteron energy of 13 MeV. The energy distribution is broad and extends from about 0.1 to 10 MeV [18]. The energy spectrum is shown in Fig. 1. The 0-degree, “free-in-air”, tissue-kerma-averaged mean neutron energy is <*E*_n_ > = 5.8 MeV [19] (for ionising radiation, kerma is defined as the sum of the initial kinetic energies of all charged particles liberated in a given mass of material by the incident uncharged particles, divided by such mass; the unit of kerma is Gy [20]). Dose to tissue was determined using a calibrated tissue-equivalent ionisation chamber according to ICRU89 [21]. The relative standard uncertainty for the total dose determination was 6%. The dose due to photon radiation was about 2.5%. Neutron doses of 0.1, 0.25, 0.5, 1 and 2 Gy were applied.

Secondly, “low-energy” monoenergetic neutrons with an energy of 1.2 MeV (0.003 Gy/min) were produced by the T(p,n)^3^He reaction and neutrons of 0.56 MeV (0.0045 Gy/min) by the ^7^Li(p,n)^7^Be reaction [22, 23]. Both energy spectra are shown in Fig. 1. The neutron yield per unit target charge at an emission angle of 0° was measured using a Long Counter. Monte Carlo neutron transport calculations were carried out to determine the spectral neutron distribution in the liquid cell suspensions from the 0° neutron yield and the known angular distribution of neutron yield and neutron energy at larger emission angles. The tissue kerma was calculated from the spectral fluence using the fluence-to-kerma conversion coefficients [19]. In this way, the contribution of neutrons scattered in the sample holder was properly accounted for. The relative contributions of scattered neutrons to the kerma and the kerma-weighted mean energy of the scattered neutrons are summarised in Table 1. The relative standard uncertainty for the total dose determination was about 7%. The dominant contribution resulted from the uncertainty of the distance of the sample to the neutron source. Cells were exposed to neutrons of 0.56 MeV with doses of 0.16 Gy, 0.40 Gy and 0.84 Gy, and to neutrons of 1.2 MeV with doses of 0.18, 0.42 and 0.85 Gy for colony forming and γH2AX assay, as well as doses of 0.82 Gy (for 0.56 MeV) and 0.88 Gy (for 1.2 MeV) for spheres formation assay.

**Table 1:** Neutron contribution for 1.2 MeV and 0.56 MeV monoenergetic neutrons

1.2 MeV				
*d*/cm	*K*_sc_/*K*_dir_	<*E*_dir_>/MeV	<*E*_sc_>/MeV	<*E*_tot_>/MeV
5.27	0.127	1.190	0.821	1.149
7.27	0.226	1.195	0.842	1.130
10.27	0.253	1.197	0.881	1.134
0.56 MeV				
*d*/cm	*K*_sc_/*K*_dir_	<*E*_dir_>/MeV	<*E*_sc_>/MeV	<*E*_tot_>/MeV
5.27	0.174	0.562	0.386	0.536
7.27	0.332	0.563	0.394	0.521
10.27	0.378	0.564	0.411	0.522

Relative contribution of scattered neutrons to the kerma *K* and the kerma-weighted mean energies <*E* > of the scattered neutrons and direct for 1.2 MeV and 0.56 MeV monoenergetic neutrons. The subscripts ‘sc’ and ‘dir’ denote scattered and direct, i.e. uncollided, neutrons, respectively. The distance of the centre of the volume containing the cell suspension from the neutron source is denoted by *d*

In order to generate a neutron spectrum similar to that produced during proton therapy, additional irradiations were performed at the KVI-CART. An uncollimated pencil beam of 190 MeV protons with a width (1σ) of 4 mm and an RMS energy spread of about 0.2% was directed onto a 300 mm cubic water phantom (with front and back layers of 8 mm PMMA) in which the protons were stopped. The beam profile at the entrance of the phantom was measured with Gafchromic EBT film. The proton current impinging on the water phantom was monitored using an ionisation chamber which was calibrated using a scintillation detector to determine the number of protons as function of the accumulated charge from the ionisation chamber. The absolute uncertainty in the number of protons entering the water phantom is estimated to be of the order of 1%. This uncertainty is mainly due to the uncertainty in the determination of the calibration factor converting the accumulated charge from the ionization chamber to the number of protons entering the water phantom. Samples were positioned behind the water phantom (at 0° relative to the incident proton beam) at a distance of 50 mm. Proton interactions in water generated a mixed gamma – secondary neutron field at the sample positions. The total dose on the sample delivered by the mixed field was determined using a Monte Carlo simulation described below to be 4.0E-15 Gy/proton. Four sets of samples were irradiated with respectively 3.80E13; 9.50E13; 1.90E14 and 3.80E14 protons entering the water phantom, with total doses of 0.152, 0.38, 0.76 and 1.52 Gy, respectively. The dose rate was chosen such that each irradiation had equal duration (5.5 h), and that such duration was comparable to that for LDR irradiations at PTB, in view of the final data comparison. The relative standard uncertainty for the total dose determination was about 5–6%.

All radiation fields and sample exposures were simulated using the Monte Carlo radiation-transport code PHITS ver. 2.88 [24], verifying dose homogeneity in the containers, dose-distance relationships and characteristics of the neutron/photon field at the container location. For the irradiation setup at KVI-CART, the primary proton beam source of energy 190 MeV was modelled as a Gaussian distribution in x-y plane with full width at half maximum (FWHM) of 0.9 cm. The energy spectrum (Fig. 1) of the secondary neutron field produced by a 190 MeV proton beam impinging on a water phantom was simulated exactly at the cell position. The dose-averaged mean neutron energy at the cell position was calculated as <*E*_n_ > =70.5 MeV. The ratio of neutron dose/total dose was 0.65, meaning 35% extra dose to the samples from gammas. This estimation of the neutron absorbed dose is done by tracking the recoil particles directly, and running PHITS in the mode that scores the energy loss of charged particles and nuclei. For neutron induced reactions below 20 MeV, PHITS was run in the Event Generator Mode using the Evaluated Nuclear Data libraries JENDL-4.0. [25]. For higher energy neutrons (and for other hadrons), the intra-nuclear cascade model INCL4.6 [26] was employed for simulating the dynamic stage of hadron-induced nuclear reactions. The quantum molecular dynamics model JQMD [27] was employed for nucleus-induced reactions. The evaporation and fission model GEM [28] was adopted for simulating the static stage for both hadron- and nucleus-induced reactions.

### Colony forming assay

Twenty-four hours after IR, 1 × 10^3^ cells were seeded in a 25 cm^2^ cell culture flask in triplicates for each dose value. Eight days later the colonies were fixed with 70% ethanol for 10 min and stained for 5–10 min with 1% crystal violet solution (Serva Electrophoresis GmbH, Heidelberg, Germany). Colonies consisting of 50 cells and more were counted. Plating efficiency and survival fractions (SF) were determined and RBE values for a survival of 10%, referred to as RBE_(SF 0.1)_ in the text, were calculated with respect to X-rays (LDR) as described by Paganetti [29].

### Immunostaining of DSBs via γH2AX antibody

Directly after irradiation, 1 × 10^4^ cells per well (1.8 cm^2^) were seeded in duplicate in chamber slides (LabTek®, Nunc, Roskilde, Denmark) and incubated for 24 h. After fixation with 2% formaldehyde and permeabilisation with 0.25% triton-X 100 (both Sigma Aldrich Chemie GmbH, Munich, Germany) the cells were consecutively incubated 60 min with anti-γH2AX antibody (1:500, clone JBW301, Merck Millipore) and Alexa Fluor 594 goat anti-mouse IgG1 (1:400, Molecular Probes®/Life Technologies, Darmstadt, Germany) for 30 min. The slides were mounted with Vectashield® containing anti-4′,6-diamidino-2-phenylindole (DAPI; Vector Laboratories, Inc., Burlingame, CA). The foci were visualised with an Eclipse TE300 inverted microscope (Nikon, Tokyo, Japan). At a magnification of 1000×, the foci of 50 cells per chamber were counted; two chambers per irradiation. The extra yield (∆Y) was calculated as the difference between irradiated samples and the individual 0 Gy control value of residual foci as a function of dose and plotted in a graph. Linearisation was performed as described by Barendsen [30]. RBE values, referred to as RBE_(foci 24 h)_ in the text, were calculated with respect to LDR X-rays via the α value with reference to Franken et al. [31, 32]. Fits to the data points using the eq. F(D) = αD + βD^2^ yielded β values of zero.

### Sphere formation assay

Twenty four hour after IR 1 × 10^4^ cells per well were plated in triplicates in ultra-low attachment 6-well plates (Corning® Costar®, Corning Incorporated, VWR, Darmstadt, Germany) for each irradiation dose. Since the cells can not adhere to the cell culture surface, they are able to form three-dimensional spheres. All samples were incubated under standard cell culture conditions. The number of spheres was counted by microscopy with a magnification of 100× at day 1–7 after seeding, which is day 2–8 after IR. The sham irradiated control (0 Gy) of each radiation quality was set to 100% at every counting day. The radiation-induced change in the number of the spheres was related to the appropriate 0 Gy control (100%).

### Statistical analysis

Data of at least three independent experiments are represented as mean values ± standard error of the mean (SEM). For the clonogenic survival and for DNA DSBs, the assessment of statistical significance of differences was performed by student t-test. The statistical analyses of all values refer to LDR X-rays. The survival−/foci-values obtained from fits to the data points were used for statistical analyses between 0 and 2 Gy. A value of *p* < 0.05 was considered to indicate a statistically significant difference. F spheres formation, the statistical significance to the individual sham-irradiated control (0 Gy) of each radiation quality was calculated via one-sample t-test and a value of *p* < 0.02 indicated a statistically significant difference.

## Results

### Clonogenic survival after IR

**Fig. 2:**
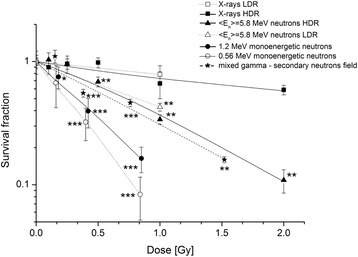
Clonogenic survival of MCF10A cells after irradiation. Data from three (LDR < *E*_n_ > = 5.8 MeV neutrons, HDR and LDR X-rays, mixed gamma – secondary neutron <*E*_n_ > = 70.5 MeV field), four (HDR < *E*_n_ > = 5.8 MeV neutrons, 0.56 MeV monoenergetic neutrons), and five (1.2 MeV monoenergetic neutrons) independent experiments, are presented as mean values ± SEM of the survival fraction. The significances refer to the equal doses of X-rays LDR irradiation. Asterisks illustrate significances: *p < 0.05, **p < 0.01, ***p < 0.001. (HDR, high dose rate; LDR, low dose rate)

**Fig. 3:**
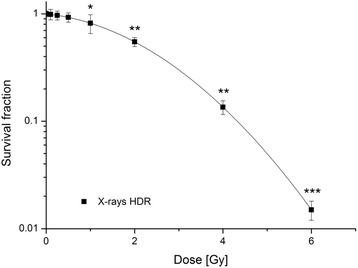
Clonogenic survival of MCF10A cells after HDR X-rays. Data from three independent experiments are presented as mean values ± SEM of the survival fraction. The significances refer to the 0 Gy control of X-rays HDR. Asterisks illustrate significances: *p < 0.05, **p < 0.01, ***p < 0.001. (HDR, high dose rate)

Long-term effects after radiation were investigated via the clonogenic survival assay. For all radiation qualities a dose-dependent decrease in the SF (Fig. 2) was observed. Additional measurements up to 6 Gy were carried out for X-rays in order to verify the linear-quadratic relation between dose and cell survival (Fig. 3). The SF after HDR X-ray irradiation was about 67% after 1 Gy and 58% after 2 Gy; 1 Gy of X-rays LDR resulted only in a small decrease of SF (78%). The strongest effects on the SF were observed after irradiations with low-energy monoenergetic neutrons of 0.56 MeV and 1.2 MeV. There were significant changes for both monoenergetic neutron energies at 0.40 Gy (for 0.56 MeV neutrons) and 0.42 Gy (for 1.2 MeV neutrons), respectively, as well as at 0.84 Gy and 0.85 Gy (0.56 MeV; 1.2 MeV) compared to the 1 Gy of LDR X-rays (SF of 78%). The effect of HDR < *E*_n_ > =5.8 MeV neutrons was slightly less pronounced: after a dose of 1 Gy the SF was 35%. This effect was still significant compared to the survival after 1 Gy of LDR X-rays. With respect to 2 Gy of HDR X-rays, the SF was significantly decreased by a factor of 5 after 2 Gy of HDR medium-energy neutrons (<*E*_n_ > =5.8 MeV). The effectiveness of 1 Gy of LDR medium-energy neutrons was higher compared to X-ray LDR as the SF was only 42%. The mixed gamma - secondary neutrons had a comparable effect on the cells as HDR and LDR medium-energy neutrons (<*E*_n_ > =5.8 MeV). After an IR of 1.52 Gy the SF was reduced to 20%. RBE values were calculated using 220 kV X-rays LDR as a reference (Table 2). Monoenergetic neutrons of 0.56 MeV and 1.2 MeV had the highest RBE values of 4.97 and 3.75 respectively. The RBE value of 2.09 for the mixed gamma - secondary neutrons (<*E*_n_ > = 70.5 MeV) is similar to RBE values of 2.06 and 1.99 for HDR and LDR medium-energy neutrons (<*E*_n_ > =5.8 MeV).

**Table 2:** Values of the α-component of LQ model for SF and DSBs for different radiation qualities

	α values of SF	α values of foci 24 h	RBE_(SF 0.1)_	RBE_(foci 24 h)_
220 kV X-rays LDR, reference	0.12	0.84	1.00	1.00
220 kV X-rays HDR	0.24	1.87	0.58	2.22
<*E*_n_ > = 5.8 MeV neutrons LDR	0.43	4.43	2.06	5.25
<*E*_n_ > = 5.8 MeV neutrons HDR	0.65	3.86	1.99	4.57
1.2 MeV monoenergetic neutrons	1.91	3.36	3.75	3.98
0.56 MeV monoenergetic neutrons	2.55	6.71	4.97	7.95
mixed gamma – secondary neutron (<*E*_n_ > = 70.5 MeV) field	1.03	3.77	2.09	4.47

RBE_(SF 0.1)_ are calculated for a survival fraction of 10%, RBE_(foci 24 h)_ are α-based calculated according to F(D) = αD + βD^2^, with β equal to zero [31, 32]

### Residual γH2AX foci induction by IR

**Fig. 4:**
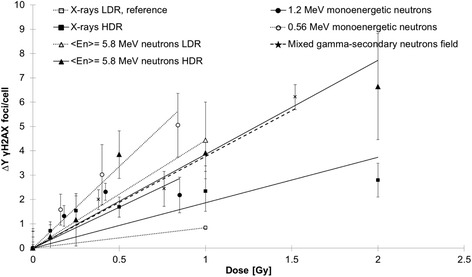
DNA double-strand breaks 24 h after irradiation. Extra yield ∆Y (difference over the individual 0 Gy control value) of residual γH2AX foci as a function of dose scored in MCF10A cells 24 h after radiation. Data from three (mixed gamma – secondary neutron <*E*_n_ > = 70.5 MeV field and X-rays) and four (medium-energy <*E*_n_ > = 5.8 MeV neutrons, 1.2 MeV and 0.56 MeV monoenergetic neutrons) independent experiments are presented as mean values ± SEM. Fitted with LQ model F(D) = αD + βD^2^, with β equal to zero [30–32]. The significances refer to the equal doses of X-rays LDR irradiation. Asterisks illustrate significances: *p < 0.05, ***p < 0.001. (HDR, high dose rate; LDR, low dose rate)

Radiation-induced residual γH2AX foci, used as markers for complex DSBs, were detected 24 h following irradiations, when repair of less complex lesions is supposed to be completed and a higher complexity of residual lesions can be assumed. The extra yield of foci was plotted with respect to sham conditions of each radiation type. The number of foci increased with higher doses for all radiation qualities (Fig. 4). Increasing doses (HDR and LDR) of 220 kV X-rays caused low but significant increases in the mean number of DSBs within the cell nuclei. Monoenergetic neutrons of 1.2 MeV as well as HDR and LDR medium-energy neutrons produced much more damage in terms of DSBs as LDR and HDR X-rays. The exposure to a mixed gamma - neutron field with secondary neutrons of <*E*_n_ > = 70.5 MeV, as performed at KVI-CART, induced an almost similar effect as HDR neutrons of <*E*_n_ > = 5.8 MeV. The α-based RBE values for foci induction showed a very clear increase following neutron radiation exposure with HDR and LDR medium-energy neutrons, low-energy neutrons of 1.2 MeV and the mixed gamma - secondary neutron field produced by a 190 MeV proton beam, when compared to X-rays. The values were even higher when the cells were irradiated with monoenergetic neutrons of 0.56 MeV (Table 2).

### Formation of spheres after IR

**Fig. 5:**
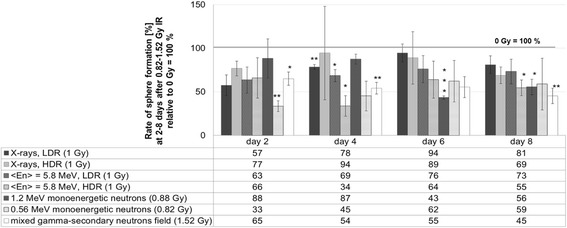
Sphere formation after irradiation. Change of the sphere formation ability of MCF10A cells at different time points after exposure to X-rays and neutrons with respect to individual 0 Gy control per day (=100% baseline). Data from three independent experiments are presented as mean values ± SEM. Significances refer to the individual 0 Gy control of each radiation quality per day. Asterisks illustrate significances: *p < 0.02, **p < 0.01, ***p < 0.002. (HDR, high dose rate; LDR, low dose rate)

The influence of IR on the sphere formation ability of human mammary epithelial cells was examined using the sphere formation assay. This assay was performed 24 h after irradiation (Fig. 5). For low-energy neutrons and for the mixed gamma – secondary neutron field the values for the highest radiation dose (0.88 Gy for 1.2 MeV neutrons, 0.82 Gy for 0.56 MeV neutrons, 1.52 Gy for the mixed field) and for medium-energy neutrons and X-rays the values for 1 Gy were normalized to the 0 Gy control, which was set to 100% for each day (see 100% baseline in the graph).

With respect to the individual 0 Gy control per day, there is a general decrease of the sphere formation ability visible for every radiation quality. The irradiation with LDR X-rays showed a clear reduction of the sphere formation ability at day 2 and 4, unlike HDR-X-rays, where no significant impairments could be observed. The exposure to neutrons caused significant changes. The use of HDR neutrons (<*E*_n_ > = 5.8 MeV) showed a slightly stronger effect, especially on day 4, as the LDR neutrons, which showed a uniform reduction at all days, which is in the range of 63–73%. Radiation with 0.88 Gy of 1.2 MeV monoenergetic neutrons and 1.52 Gy of a mixed gamma-secondary neutron field showed time-dependent a reducing effect on the sphere formation ability. On the eighth day, the ability to form spheres is more restricted than on the second day after irradiation. In addition, the exposure to 0.56 MeV monoenergetic neutrons resulted in a strong reduction of the sphere formation ability already 2 days after irradiation but this decrease seemed to recover within the following 6 days.

## Discussion

In order to investigate the RBE of neutrons relative to photons as a function of neutron energy, the present study examined the different radiobiological effects following neutron and X-ray radiation. We also measured the RBE of a mixed gamma - secondary neutron field, generated by interactions of 190 MeV protons in water, with a neutron dose equal to 65% of the total dose, and neutrons with an energy spectrum similar to that produced in a clinical setting for proton therapy. Currently, there are limited systematic studies on the effects of neutron exposure as a function of neutron energy on normal tissue cells, highlighting the comparison of neutrons to photons (an example in this sense is the study by Göhde et al. with neutron energies of 0.56 MeV, 2.5 MeV and 14.8 MeV [33]).

Concerning clonogenic survival, our data showed a large variation between the effects of various neutron energies with regard to the use of doses up to 1 Gy. Presented results about the radiobiological effects on the clonogenic survival with low-energy monoenergetic neutrons are of particular significance in contrast to X-rays and medium-energy neutrons (<*E*_n_ > = 5.8 MeV). Compared to X-rays (LDR), the SF was significantly decreased after irradiation with monoenergetic neutrons (0.56 MeV, 1.2 MeV). This effectiveness of 0.56 MeV neutrons on the clonogenic survival confirms the results of Okumura et al. [34]. Frankenberg-Schwager et al. [22] obtained an RBE of 5.4 for a different cell line, experimental conditions and reference radiation quality. These studies confirm and support our results on the effect of neutrons, but lack the representation of human tissue specific cells, using non-human or hybrid cell lines and also a smaller range of applied doses. The present study quantifies the long-term effects of neutrons with different energies and doses on normal human tissue cells from the mammary gland.

A common feature of our study and others [22, 34] is the use of cell suspensions for the irradiation. A study investigating different cell culture models used during the irradiation was led by Cansolino and colleagues [35]. Here, the SF for cell suspension and adherent cells was determined for rat colon adenocarcinoma cell lines. In accordance to our data, they showed a higher impact of neutrons (up to 10 Gy) on suspended cells compared to ^60^Co.

Furthermore, next to energy, radiation type and doses, the dose rate can play an important role for the radiobiological effects, especially regarding the induction of residual γH2AX foci. For DNA damage, represented by γH2AX foci (DSBs) 24 h after radiation exposure, our findings reveal a 2-fold higher response of cells irradiated with HDR in contrast to LDR X-rays. This can be a result of repair processes, which start after a few minutes. Therefore, DSBs induced by LDR may already be partially repaired during the irradiation process before the full applicable dose is reached, as the time for a dose of 1 Gy generated with LDR is more than 20-times longer than generated with HDR [36].

Our results demonstrated that the number of residual γH2AX foci 24 h after IR, as an indicator for DSBs, increased as a function of increasing dose, which has been also reported by Okumura and colleagues [34], who observed 53BP1 foci as an indicator for DSBs for several time points. In contrast, they could not identify a difference between samples (1–3 h following IR) exposed to neutrons (mixed beam) and γ-rays, however, they did not examine the residual γH2AX foci 24 h after radiation. Just as Tanaka et al. observed DNA damage by means of comet assay [37], we could also demonstrate a higher biological effectiveness of 0.56 MeV monoenergetic neutrons compared to 1.2 MeV neutrons. Their analysis of DNA damage demonstrated the same pattern of effectiveness of the different radiation qualities.

Using a sphere formation assay, we examined the radiation-induced response of a potential stem-like subpopulation: it is known that MCF10A cells include a progenitor like cell subpopulation [15]. Present data showed alterations in the sphere formation ability, which were more evident following monoenergetic neutron irradiation and following the exposure to the mixed gamma - secondary neutron field, compared to X-rays and medium-energy neutrons (<*E*_n_ > = 5.8 MeV). Like Dionet et al., who investigated fast neutrons on normal skin fibroblast by cell survival assay, we could show a stronger effect following HDR neutrons compared to LDR neutrons [38]. In general, most publications concerning sphere formation combining mammary cells and radiation are dealing with cancer cell lines [39, 40]. The present study gives instead a first insight on the sphere formation ability of normal human breast cells irradiated with a broad range of neutron energies.

From the presented dataset, observable differences in RBE values by various neutron energies relative to X-rays can be concluded.

Consistent with Tanaka et al. [37] and Schmid et al. [23], we obtained results with increasing RBE values as a function of decreasing neutron energies (between 0.56 MeV and <*E*_n_ > = 5.8 MeV). The mixed gamma - secondary neutron (<*E*_n_ > = 70.5 MeV) field, generated by protons of 190 MeV impinging on a water phantom, yielded RBE values for both, SF and residual foci, which are comparable to those of HDR and LDR medium-energy neutrons of <*E*_n_ > = 5.8 MeV: 4.47 for RBE_(foci 24 h)_ and 2.09 for RBE_(SF 0.1)_. As well known, RBE is a variable function of several factors, among which the endpoint itself [37, 41]. Qualitative consistency is found in this study between RBE_(SF 0.1)_ and RBE_(foci 24 h)_, both increasing with decreasing neutron energies for the covered range from 0.56 MeV to <*E*_n_ > = 5.8 MeV.

## Conclusions

The present study extensively investigated chosen radiobiological effects following exposures in the dose range of 0 Gy up to 2 Gy to different neutron energies compared to X-rays in MCF10A normal human breast cells. The range of selected neutron energies (0.56 MeV, 1.2 MeV and a broad spectrum with a mean energy of 5.8 MeV) was expanded by the use of a mixed gamma - secondary neutron field (<*E*_n_ > = 70.5 MeV), adopted to simulate the scattered neutron field during proton therapy. Dose-rate effects were also addressed when high vs. low dose rate exposures could be performed (X-rays and medium-energy neutron exposure). Effects on clonogenic survival and γH2AX residual foci induction are reported, which were strongly dependent on radiation quality and dose (dose rate for residual foci induction only). RBE values were extracted from measured endpoints as RBE_(SF 0.1)_ and RBE_(foci 24 h)_. They are found to be coherently increasing for decreasing neutron energy in the investigated energy range. The exposure to the mixed gamma - secondary neutron field yield RBEs as high as for medium-energy neutrons. The response of the potential fraction of stem-like cells in the MCF10A cell population was also addressed, by measuring sphere formation ability for up to 8 days after exposure with the maximal dose or 1 Gy for each radiation quality (0.88 Gy – 1.52 Gy).This investigation provides a deeper insight into the radiobiological effects of neutron exposure, which is very important in order to assess the risk of secondary neutrons produced during conventional and particle radiotherapy and their possible trigger function for potential carcinogenic effects on normal breast cells and stem cells.

## Additional file

Additional file 1: Authentification certification of MCF10A cell line. (PDF 1337 kb)

## Abbreviations

BSA: Bovine serum albumin
CO_2_: Carbon dioxide
DAPI: 4′,6-diamidino-2-phenylindole
DMEM: Dulbecco’s modified Eagle medium
DSB: Double strand break
EGF: Epidermal growth factor
HDR: High dose rate
IR: Ionising radiation
LDR: Low dose rate
PBS: Phosphate buffered saline
PMMA: Polymethylmethacrylate
RBE: Relative biological effectiveness
SF: Survival fraction
γH2AX: Phosphorylated histone 2AX
