# Supplementary material for: Comparative study of the effects of different radiation qualities on normal human breast cells
Source: Radiat Oncol. 2017 Sep 25;12:159. doi: 10.1186/s13014-017-0895-8 (PMC5613446; doi:10.1186/s13014-017-0895-8)

Leibniz-Institut DSMZ-Deutsche Sammlung von Mikroorganismen und Zellkulturen GmbH

Leibniz-Institut DSMZ GmbH · Inhoffenstraße 7 B · 38124 Braunschweig

Inhoffenstraße 7 B
38124 Braunschweig
GERMANY

Tel. +49(0)531 26 16-166
Fax: +49(0)531 26 16-150
E-Mail: wdi@dsmz.de
Internet: www.dsmz.de

Frau Dr. Katrin Manda
Universtäsmedizin Rostock
Klinik und Poliklinik für Strahlentherapie
Strahlenbiologisches Labor
Südring 75
18059 Rostock

Ihr Zeichen/Your ref. Unser Zeichen/Our ref.wdi +49(0)531-2616-166 Datum/Date 10.03.2015

Sehr geehrte Frau Dr. Manda,

vielen Dank für Ihren Auftrag zur Identifizierung von Zelllinien. Wir haben von Ihrer Probe mit Hilfe einer nonaplex PCR ein DNA Profil von 8 hoch polymorphen Orten von Short Tandem Repeats (STRs) hergestellt. Zusätzlich wurde die humane Probe auf Anwesenheit von DNA Sequenzen aus Maus, Ratte und Hamster getestet. Der Abgleich des Profils mit unserer Datenbank brachte folgendes Ergebnis:

#	sample	parental/reference line	comment/match
1	MCF10A	MCF 10A (ATCC CRL-10317)	full-matching STR reference STR profile of MCF 10A, authentic

Die Zellprobe zeigt ein heterozygotes humanes DNA Profil und ist mit einer Nachweisgrenze von 10^{-5} frei von Animalzellen aus Maus, Ratte, chinesischem und syrischem Hamster. Die Probe entstammt einer rein humanen Zellkultur.

Das STR Profil der Probe MCF 10A zeigt eine vollständige Übereinstimmung mit dem STR Profil der parentalen Zelllinie MCF 10A der internationalen STR Referenzdatenbank der Zellbanken ATCC (USA), JCRB/RIKEN (Japan), HPACC (UK), KCLB (Korea) und der DSMZ auf. Die Probe MCF 10A wurde einer authentischen Zellkultur entnommen.

Auf Grund der Exklusionsrate des genutzten STR Systems mit einer Wahrscheinlichkeit von 1 in 114.000.000 sind die Ergebnisse als sicher zu betrachten. Bitte finden Sie die Dokumentation der Analyse (Elektropherogramme) und eine Tabelle mit der Allelliste im Anhang.

Mit freundlichen Grüßen,
W. Dirks

Geschäftsführer/
Managing Director:
Prof. Dr. Jörg Overmann
Aufsichtsratsvorsitzender/Head of
Supervisory Board: MR Dr. Axel Kollatschny

Braunschweigische Landessparkasse
Kto.-Nr./Account: 2 039 220
BLZ/Bank Code: 250 500 00
IBAN DE22 2505 0000 0002 0392 20
SWIFT (BIC) NOLADE 2 H

Handelsregister/
Commercial Register:
Amtsgericht Braunschweig
HRB 2570
Steuer-Nr. 13/200/24030

Zertifikat seit 2010 audit berufundfamilie

Quality Management ISO 9001:2008

	Cell line	Date	D5	D5'	D13	D13'	D7	D7'	D16	D16'	vWA	vWA'	TH01	TH01'	TPOX	TPOX'	CSF1	CSF1'	Amel	Amel'	Date Animal-PCR:	M	R	CH	SH
ATCC (CRL-10317)	MCF 10A		10	13	8	9	10	11	11	12	15	17	8	9.3	9	11	10	12	X	X					
Manda, Universitätsmedizin Rostock, Klinik und Poliklinik für Strahlentherapie, Strahlenbiologisches Labor	MCF10A	10.03.2015	10	13	8	9	10	11	11	12	15	17	8	9,3	9	11	10	12	X	X	10.03.2015	-	-	-	-

STR-profile of reference

STR-profile of analysed sample

MCF10A.C01_150310131C

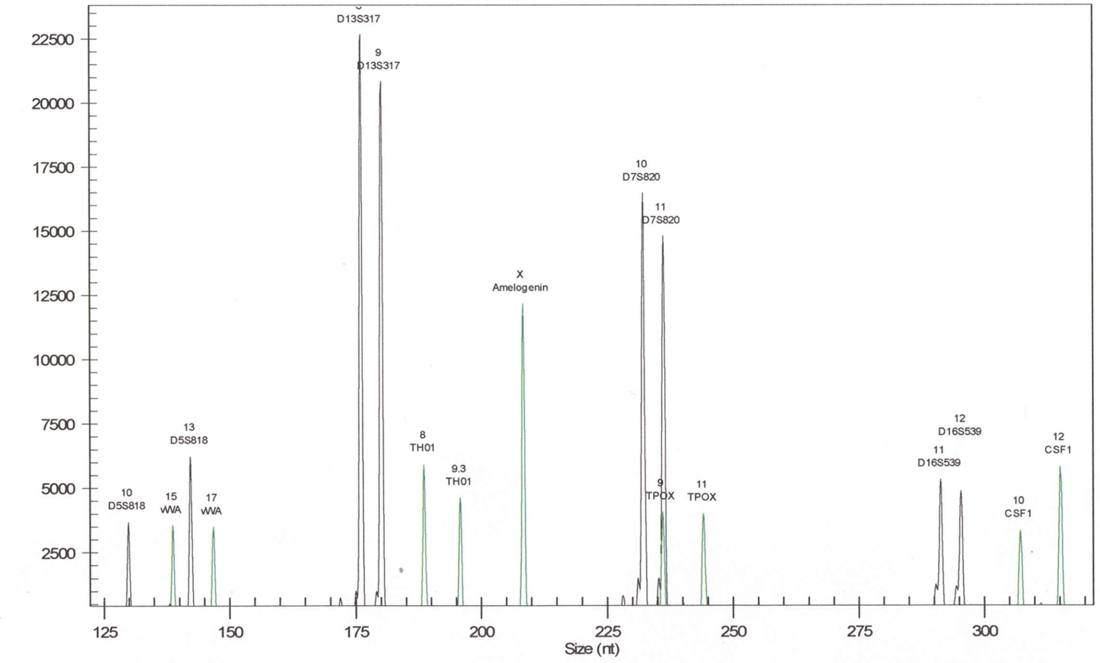

Supplement: Additional file 1: — Authentification certification of MCF10A cell line. (PDF 1337 kb) [file 13014_2017_895_MOESM1_ESM.pdf]
